# Detection of Dopamine Based on Aptamer-Modified Graphene Microelectrode

**DOI:** 10.3390/s24092934

**Published:** 2024-05-05

**Authors:** Cuicui Zhang, Tianyou Chen, Yiran Ying, Jing Wu

**Affiliations:** School of Science, China University of Geosciences (Beijing), Beijing 100083, China; 2019220038@email.cugb.edu.cn (C.Z.); 3019210007@email.cugb.edu.cn (T.C.); yyiran@email.cugb.edu.cn (Y.Y.)

**Keywords:** graphene fibers, microelectrodes, aptamers, electrochemistry, specific recognition

## Abstract

In this paper, a novel aptamer-modified nitrogen-doped graphene microelectrode (Apt-Au-N-RGOF) was fabricated and used to specifically identify and detect dopamine (DA). During the synthetic process, gold nanoparticles were loaded onto the active sites of nitrogen-doped graphene fibers. Then, aptamers were modified on the microelectrode depending on Au-S bonds to prepare Apt-Au-N-RGOF. The prepared microelectrode can specifically identify DA, avoiding interference with other molecules and improving its selectivity. Compared with the N-RGOF microelectrode, the Apt-Au-N-RGOF microelectrode exhibited higher sensitivity, a lower detection limit (0.5 μM), and a wider linear range (1~100 μM) and could be applied in electrochemical analysis fields.

## 1. Introduction

Neurotransmitters (NTs) are signaling substances that can control human physiological activities by regulating communication within neural networks. NTs can affect many neurophysiological and psychological functions, such as sleep, learning, memory, and mood [1]. Dopamine (DA) is one of the most crucial neurotransmitters in the central nervous system, and it is associated with human desires and emotions. It plays a fundamental role in transmitting messages of excitement and pleasure. Abnormal levels of DA in the body can lead to various diseases, such as Parkinson’s disease, schizophrenia, Tourette syndrome, attention deficit hyperactivity disorder, and pituitary tumors, among other neurological disorders [2,3,4]. Therefore, the accurate detection of this biomolecule is crucial.

Among the techniques used for detecting low concentrations of DA, electrochemical analysis stands out for its high sensitivity, fast response time, practicality, low cost, and ease of operation [5]. This can be attributed to the flexible design of electrode materials and the easy oxidation characteristics of the phenolic hydroxyl functional groups in DA [6]. The oxidation mechanism of dopamine involves a two-electron and two-proton transfer process where dopamine is oxidized to form dopamine quinone (DAQ). As the electrode accepts electrons from the oxidized dopamine molecules, it generates a Faradaic current signal that can be measured [7].

However, the electrochemical signal is weak when the conventional electrode is used to detect DA because of the slow electron transfer rate on the electrode surface. The electrode is modified by coating different materials, thereby overcoming the problem of a weak current signal and sensitively and rapidly detecting DA [8], such as carbon nanomaterials (including carbon black, graphene oxide, carbon quantum dots, and graphene quantum dots) [9], polymers [10], metal oxides (CuO, MnO_2_) [11,12], metal nanoparticles (Au, Pd, Cu) [13,14,15], MOF materials [16,17], and so on.

In electrochemical sensing, microelectrodes can also be used in addition to modifying conventional electrodes to improve their sensitivity to DA. As electrochemical working microelectrodes, they are usually made of platinum, gold, silver, and other metal materials or carbon-based fiber materials, and their one-dimensional size is micron or nanometer. Because of their miniature size, the surface diffusion process of microelectrodes is similar to that of spherical electrodes, so it shows unique electrochemical characteristics. Due to their high stability, high resolution, high mass transfer efficiency, and rapid response, microelectrode electrochemical detection has become the main means of detecting biomolecules and recording neural signals [18,19,20]. Metal-based microelectrodes have good catalytic performance, but they have the disadvantages of general flexibility, heavy weight, and easy oxidizability, and the mechanical mismatch between metal-based microelectrodes and surrounding tissues may cause an inflammatory response. Carbon-based materials have the advantages of excellent conductivity, low cost, and high stability. Among carbon-based materials, carbon-based fiber materials have the characteristics of a large specific surface area, high flexibility, easy functionalization, good biocompatibility, and stable electrochemical performance, which provide abundant active sites for improving sensitivity and exerting an excellent electrocatalytic effect [3,21].

Here are some examples of carbon-based fiber electrodes utilized for DA detection: carbon fiber microelectrodes [22], carbon nanotube fiber microelectrodes [23], and graphene microelectrodes [24]. Carbon-based fiber electrodes eliminate any post-treatment or modification typically required for the preparation of DA sensors, such as electrode oxidation or Nafion coating [25]. Graphene fibers indeed possess several advantages over carbon fiber and carbon nanotube fiber electrodes that make them particularly suitable for the sensitive and selective electrochemical sensing of dopamine [26]. While graphene fiber microelectrodes offer numerous advantages for dopamine detection, their native performance may not always meet the stringent requirements for sensitive and selective measurements in complex biological environments. To address this question, researchers often employ various strategies to enhance the detection capabilities of these electrodes [27,28]. Graphene oxide (GO) is a derivative of graphene that contains a variety of oxygen-containing functional groups such as hydroxyl, epoxy, and carboxyl moieties attached to its basal planes and edges. These functional groups make GO highly hydrophilic and chemically reactive, which are key properties for the further modification and fabrication of reduced graphene oxide (RGO) with tailored characteristics for specific applications, including electrochemical sensing [29]. The nitrogen doping of reduced graphene oxide fibers (N-RGOF) has indeed become a prominent strategy for enhancing the performance of graphene-based materials, especially in the fields of electrocatalysis and biosensing. Nitrogen atoms are similar in size to carbon atoms and provide five valence electrons to combine with carbon atoms, which changes the internal electronic structure of graphene, plays the role of active sites, and improves the electrocatalytic activity. Therefore, N-RGOF is widely used in electrocatalysis and biosensors [30,31,32]. Although N-RGOF microelectrodes possess excellent conductivity, stability, and rapid response, it is still necessary to further enhance the selectivity of microelectrodes for precise measurement in complex biological systems, enabling them to specifically target biomolecules. Chemical modification of the microelectrode surface with target recognition ligands is an attractive strategy to improve the selectivity of microelectrodes [33]. For example, Li [34] et al. constructed a novel electrochemical sensor using Au NPs and poly (o-aminothiophenol) (oATP) film to modify the GCE. The Au NPs enhanced the electrode conductivity and facilitated electron transfer, endowing the sensor with higher analytical sensitivity and selectivity. It enabled the ultrasensitive and specific detection of low-density lipoprotein in human serum samples, with a detection limit of 3.25 ng/mL. This sensor offers a novel and promising method for clinical LDL level detection. Ferlazzo [35] et al. used N-succinimidyl ester (DSP) as a linker, which forms a thiol bond with the gold electrode and an amide bond with the amino group on Phe dehydrogenase (PHD). PHD can effectively convert phenylalanine (Phe) into phenylpyruvate. This potentiometric sensor utilizes an Au electrode loaded with a linker to covalently attach PHD for monitoring Phe in phenylketonuria (PKU) patients. Patil [36] et al. developed a flexible, disposable gold nanoparticle-N-doped carbon-modified electrochemical sensor that could simultaneously detect DA and UA. However, its detection range and selectivity for DA were relatively low.

Antigen–antibody-specific recognition has become one of the most classic strategies used to improve selectivity. However, the inactivation of proteins in harsh environments leads to the failure of this strategy. Aptamers have the advantages of low immunogenicity, easy chemical modification, good physical stability, and low cost of bulk synthesis [37,38]. These characteristics have attracted widespread attention in the field of analysis, and more and more researchers tend to modify aptamers on electronic sensing platforms, achieving considerable results [39].

In this experiment, Au NPs were utilized to load onto the surface of a fiber electrode to enhance its conductivity and facilitate electron transfer. They also bonded with the aptamer through Au-S bonds to selectively recognize DA. In addition, by loading aptamers that selectively recognize DA, the detection range was enhanced. In this study, we report a simple two-step strategy for the aptamer modification of nitrogen-doped reduced graphene oxide fiber microelectrodes (Apt-Au-N-RGOF). The research shows that gold nanoparticles adsorbed on the surface defects of N-RGOF can effectively immobilize aptamers on the fiber surface through Au-S bond self-assembly, endowing the fiber with certain surface selectivity when in contact with biomolecules [40,41]. Electrochemical characterization tests show that Apt-Au-N-RGOF has higher sensitivity and a lower detection limit for DA compared to N-RGOF. Apt-Au-N-RGOF has a wide linear response to DA in the range of 1 μM to 100 μM and a detection limit of 0.5 μM and could be applied in electrochemical analysis fields.

## 2. Materials and Methods

### 2.1. Materials

Graphite powder (99.95%, Shanghai Aladdin Reagent Co., Ltd., Shanghai, China); hydrochloric acid, sulfuric acid, sodium nitrate, and potassium permanganate (30%, 98%, 99%, 99%, Beijing Chemical Plant, Beijing, China); potassium ferricyanide, urea, ethanol, DA, and tetrachloroauric(III) acid trihydrate (99.5%, 99%, 99.5%, 98%, 99.9%, Beijing Innochem Technology Co., Ltd.,Beijing, China); potassium chloride and uric acid (99.5%, 99%, Beijing Oka Biotechnology Co., Ltd., Beijing, China); aptamers (Shanghai Sangon Biotech Bioengineering Co., Ltd., Shanghai, China); and 0.1 M phosphate buffered solution (PBS), which was self-made, were all used in this study. 

### 2.2. Preparation of Nitrogen Doping of Reduced Graphene Oxide Fibers

**Figure 1:**
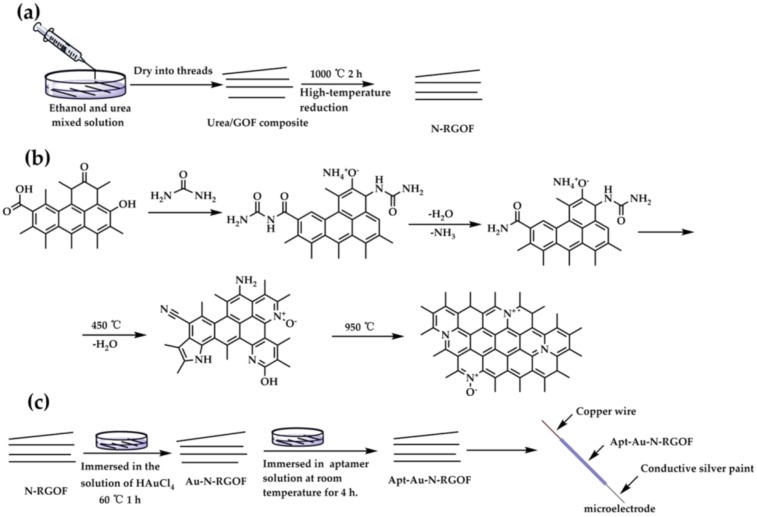
Preparation of N-doped reduced graphene fiber and microelectrode: (**a**) fiber prepared by wet spinning method; (**b**) nitrogen doping mechanism of urea; (**c**) preparation of microelectrode.

GO was prepared by the modified Hummers method [42]. N-RGOF was prepared by wet spinning, and the operation steps and mechanism are shown in Figure 1a,b. The operation was as follows: 0.5 g of urea was dissolved in 20 mL of ethanol (98%) by ultrasound to form a mixed solution, and then the concentrated GO solution (about 12 mg/mL) was slowly and uniformly wet-spun in the mixed solution of urea–ethanol with a syringe. After the urea solution was completely immersed in the fiber and soaked for several minutes, the fiber was removed from the solution. The urea/GOF was dried at room temperature. Finally, N-RGOF was prepared by annealing the urea/GOF at 1000 °C in nitrogen atmosphere for 2 h [43,44].

### 2.3. Preparation of Microelectrode

The preparation of microelectrode is shown in Figure 1c. The N-RGOF was immersed in a 10 mM aqueous solution of HAuCl_4_ for 1 h, allowing Au/Au^3+^ nanoparticles to adsorb onto the defect sites on the graphene surface. Then, the resulting material was annealed at 60 °C for 1 h to produce Au-N-RGOF. The Au-N-RGOF was washed with 10 mM PBS (phosphate-buffered saline) and deionized water to remove any Au nanoparticles that had not been adsorbed onto the fiber surface [45]. The Au-N-RGOF was incubated in a 1 μM aptamer solution prepared in 10 mM PBS at room temperature for 4 h. During this incubation, the aptamers self-assembled and immobilized on the fiber surface by forming Au-S bonds. The fiber was washed three times with 10 mM PBS solution and deionized water to remove any aptamers that had non-specifically bound to the fiber. The final product, Apt-Au-N-RGOF, was obtained. Then, the Apt-Au-N-RGOF was loaded onto the copper wire through conductive silver paint to produce the microelectrode. This product was used for various biosensing applications where the specific recognition of the target molecules was required.

### 2.4. Electrochemical Test Conditions

The scan rate of CV is 50 mV/s, and the electrolyte is 0.1 M PBS. CV and DPV measurements were performed on an electrochemical workstation (CHI 760e) consisting of a platinum (Pt) plate electrode (counter electrode), an Ag/AgCl electrode (reference electrode), and a fiber microelectrode (working electrode). The size of the Pt sheet electrode is 5 × 5 × 0.1 mm (length×width×thickness), and the Ag/AgCl electrode and the Pt sheet electrode were purchased from Tianjin Aida Hengsheng Technology Development. The amount of biosensor used was approximately 2 mm of Apt-Au-N-RGOF, which was then immersed in an electrolyte solution.

## 3. Results and Discussion

### 3.1. Structural and Morphological Characterizations of Aptamer-Modified Au-N-RGOF

**Figure 2:**
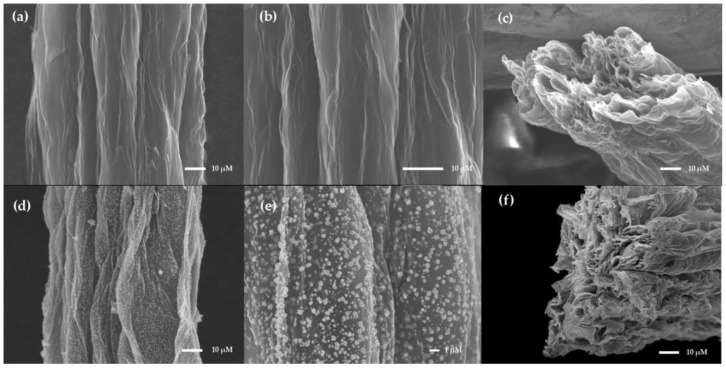
Representation SEM images of N-RGOF and Au-N-RGOF: (**a**) N-RGOF surface; (**b**) N-RGOF surface; (**c**) N-RGOF cross-section; (**d**) Au-N-RGOF surface; (**e**) Au-N-RGOF surface; (**f**) Au-N-RGOF cross-section.

The SEM images of N-RGOF and Au-N-RGOF are shown in Figure 2. The surface of N-RGOF is rough and heavily wrinkled in Figure 2a,b. In addition, it can be seen from the cross-sectional view that the N-RGOF exhibits a layered stacking structure with many hollow configurations in Figure 2c, which provides vacancies for adsorbing nanoparticles. In addition, this hollow layered structure makes N-RGOF have a larger specific surface area than other microelectrodes with the same size, which can significantly improve the mass transfer efficiency and current density of N-RGOF microelectrodes and further improve the sensitivity. Figure 2d,e show that the soaked and annealed Au nanoparticles are uniformly distributed on the surface of N-RGOF. The introduction of Au nanoparticles not only provides a large number of chemical active sites for the modification of aptamers but also improves the conductivity of N-RGOF based on its low impedance performance. The EDX quantitative data from the SEM analysis of Au-N-RGOF confirm the presence of Au components, as seen in Table 1. This table provides essential evidence to support the successful incorporation of gold nanoparticles within the nitrogen-reduced graphene oxide framework (N-RGOF).

**Figure 3:**
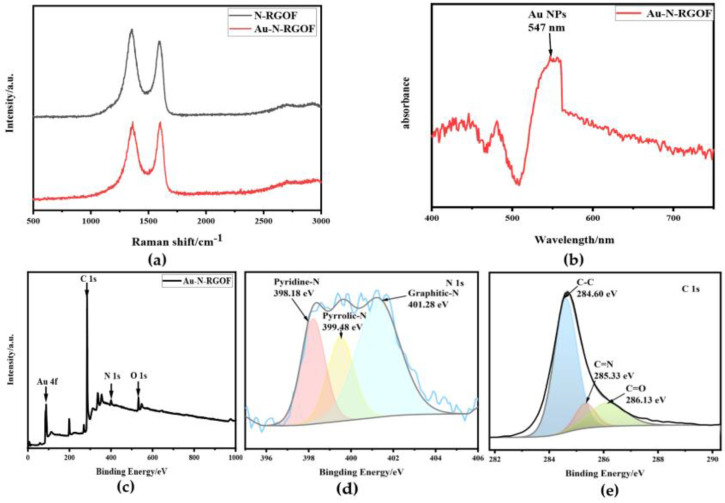
Raman spectra of N-RGOF, UV–Vis absorption spectra of Au-N-RGOF and Au-N-RGOF and XPS spectrum of Au-N-RGOF: (**a**) Raman spectra of N-RGOF and Au-N-RGOF; (**b**) UV–Visible absorption spectra of Au-N-RGOF; (**c**) XPS total spectra of Au-N-RGOF; (**d**) the corresponding high-resolution N 1s peak; (**e**) the corresponding high-resolution C 1s peak.

The Raman spectrum of the sample is shown in Figure 3a, where the D and G bands of the carbon material correspond to peaks centered at 1353 and 1591 cm^–1^ [28], respectively. The I_D_/I_G_ ratio of N-RGOF is about 1.14, and the I_D_/I_G_ ratio of Au-N-RGOF is about 1.02, which indicates that the doped gold nano-ions are loaded on the defects on the surface of the graphene [41]. From Figure 3b, the absorption peaks at 547 nm are the typical surface plasmon resonance (SPR) bands of AuNPs, confirming the successful loading of Au in the UV–Visible absorption spectra [14,46]. X-ray photoelectron spectroscopy (XPS) was used to analyze the surface elemental composition and the bonding configurations of the elements in Au-N-RGOF. The XPS total spectrum of Au-N-RGOF is shown in Figure 3c, which proves that the Au and N elements are loaded on RGOF [29,47,48]. The N 1s spectrum can be deconvoluted into peaks attributed to pyridinic-N (398.18 eV), pyrrolic-N (399.48 eV), and graphitic-N (401.28 eV) in Figure 3d. The C 1s XPS spectra after deconvoluting shows three peaks corresponding to C-C (284.6 eV), C=N (285.33 eV), and C=O (286.13 eV), respectively, from Figure 3e. This indicates that nitrogen atoms have been successfully doped into the RGOF framework, and there are three different types of nitrogen-doped structures. The successful doping of nitrogen can enhance the electrochemical behavior of RGOF, as redox reactions are prone to occur at doping sites or on adjacent carbons where the electronic structure has been altered. The above characterizations demonstrate the successful preparation of Au-N-RGOF.

### 3.2. Electrochemical Characterization and Anti-Interference of N-RGOF

**Figure 4:**
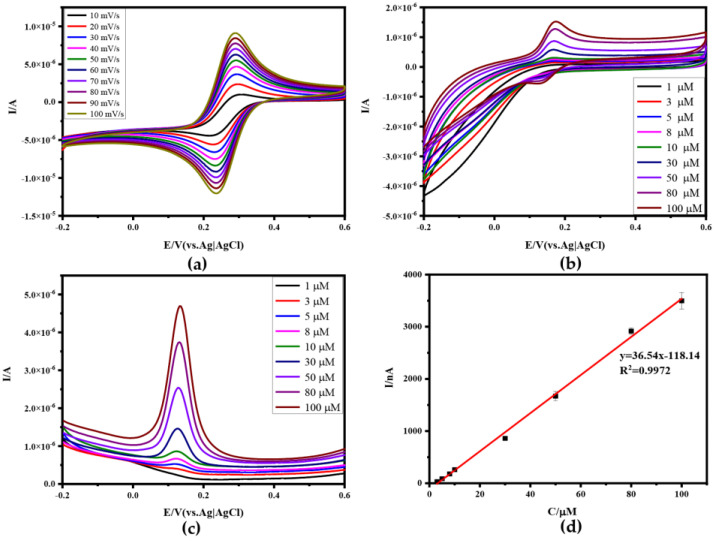
Electrochemical characterization of N-RGOF and detection of DA: (**a**) CV of N−RGOF in 0.5 M KCl with 5.0 mM K_3_Fe(CN)_6_ at different scan rates; (**b**) CV of N−RGOF in DA solution with different concentrations (1 μM, 3 μM, 5 μM, 8 μM, 10 μM, 30 μM, 50 μM, 80 μM, 100 μM); (**c**) DPVs of N−RGOF in DA solution with different concentrations; (**d**) linear relationship between DA concentration and DPV peak current.

**Figure 5:**
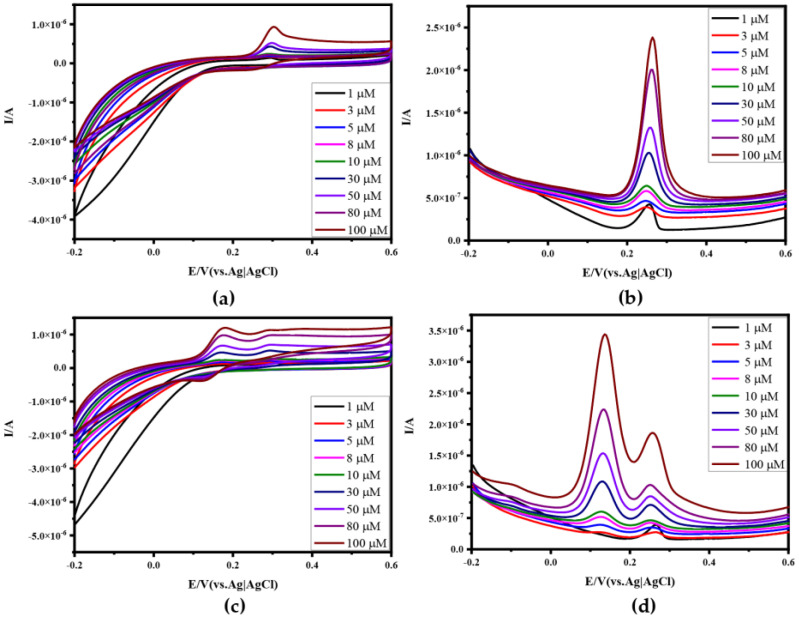
Electrochemical characterization of N−RGOF in UA solution or mixture solution of UA and DA: (**a**) CV of N−RGOF in UA solution with different concentrations (1 μM, 3 μM, 5 μM, 8 μM, 10 μM, 30 μM, 50 μM, 80 μM, 100 μM); (**b**) DPV of N−RGOF in UA solution with different concentrations; (**c**) CV of N−RGOF in mixed solution of DA and UA with different concentrations; (**d**) DPV of N−RGOF in mixed solution of DA and UA with different concentrations.

The electrochemical performance of N-RGOF was preliminarily tested in 0.5 M KCl solution containing 5.0 mM of K_3_Fe(CN)_6_ by CV plots at different scan rates in Figure 4a, and the redox peak indicates that N-RGOF has excellent electrocatalytic performance as a microelectrode. The CV of N-RGOF is shown at different concentrations of DA in 0.1 M PBS at a scan rate of 50 mV/s in Figure 4b, and the corresponding DPV is shown in Figure 4c. According to the CV and DPV curves, the oxidation peak of DA is 0.144 V, and the electrical signal of the DA oxidation peak can be detected in the range of 3 μM~100 μM, and the peak current increased linearly with the increase in the DA concentration from 3 μM to 100 μM (Figure 4d). The above data showed that N-RGOF had a linear detection range (R^2^ = 0. 9972) with high sensitivity and a low concentration, and the detection limit was 3 μM. To investigate the anti-jamming and selectivity of N-RGOF in the presence of interfering molecules, we found that the oxidation peak of UA is 0.26 V in Figure 5a,b, which is neither too close nor too far from the oxidation peak of DA. And more importantly, they do not react, which is very suitable for study as interfering biomolecules. As can be seen from Figure 5b, DPV also responded when the concentration of UA was 1 μM. Compared with DA, the detection limit of UA by N-RGOF is lower, but it is obviously not in the linear range. As shown in Figure 5c,d, the CV and DPV of the mixed solution of DA and UA at different concentrations were measured. The addition of the interfering molecule UA did not affect the detection performance of DA, and the oxidation peaks of the two molecules could be identified at the same time, indicating that N-RGOF had a certain anti-interference ability at low concentrations. However, it is not difficult to find that the baseline of DA changes gradually with the increase in the DA and UA concentrations. This is because as the concentration increases, UA begins to be oxidized before the DA oxidation peak drops to the baseline, and eventually presents a continuous oxidation peak, making it difficult to distinguish the baselines of the two biomolecules. Therefore, it is necessary to improve the selectivity of N-RGOF by loading Au nanoparticles on the surface of N-RGOF and connecting aptamers with Au-S bonds to eliminate the interference of other molecules by specific recognition of the target molecules.

### 3.3. Electrochemical Characterization and Anti-Interference of Aptamer-Modified Au-N-RGOF

**Figure 6:**
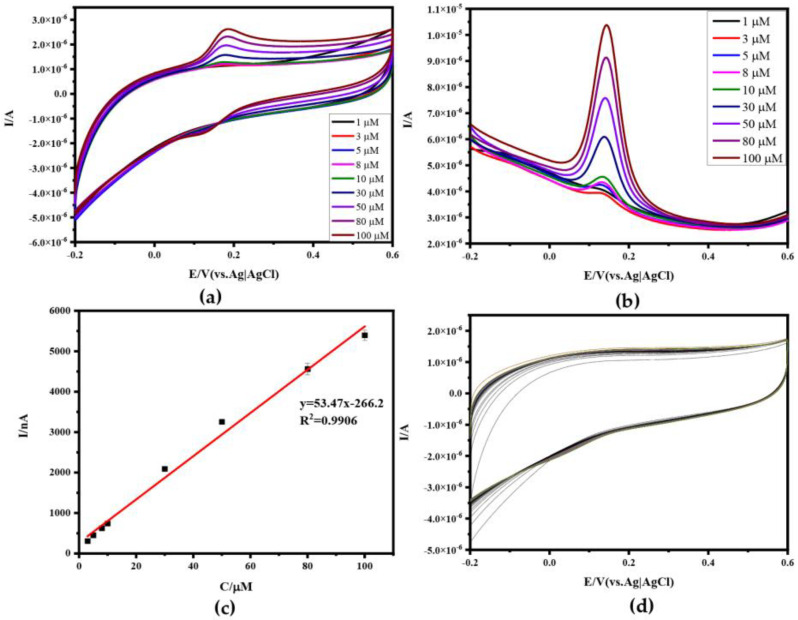
Electrochemical characterization of Apt−Au−N−RGOF in DA solution and 100 times CV cycles of RGOF in blank 0.1 M PBS solution: (**a**) CV of Apt−Au−N−RGOF in DA solution with different concentrations (1 μM, 3 μM, 5 μM, 8 μM, 10 μM, 30 μM, 50 μM, 80 μM, 100 μM); (**b**) DPV of Apt−Au−N−RGOF in DA solution with different concentrations; (**c**) linear relationship between different DA concentrations and DPV peak current; (**d**) RGOF 100 CV cycles in blank 0.1 M PBS solution.

Since Au doping reduces the impedance of N-RGOF and further improves its conductivity, more than 10 CV scans in PBS solution are required to stabilize the performance of the microelectrode, and the stability improves with the increase in the number of CV scans. The CV diagram at different concentrations of DA in 0.1 M PBS is shown in Figure 6a. The current of Apt-Au-N-RGOF is more stable than that of N-RGOF. In addition, it can be preliminarily seen that the detection limit of DA is improved to 1 μM in Figure 6b. This is due to the increased ability of the aptamer to specifically recognize and capture DA molecules. When the voltage was raised to the oxidation potential of DA (0.144 V), the electrons generated by the oxidation of DA could be transferred to the microelectrode through the aptamer, and finally they were presented in the form of the oxidation peak of DPV. In the range of 1 μM to 100 μM, the oxidation peak current increased linearly with the increase in the DA concentration in Figure 6c, and the detection sensitivity was calculated to be 58.82 nA mM^−1^ (R^2^ = 0.9981), and the LOD was calculated to be 0.5 μM by the LOD formula.

(1) $$\mathrm{LOD}=\frac{{3S}_{b}}{S}$$

where S_b_ is the standard deviation of 15 blank measurements and S is the sensitivity in the formula.

**Figure 7:**
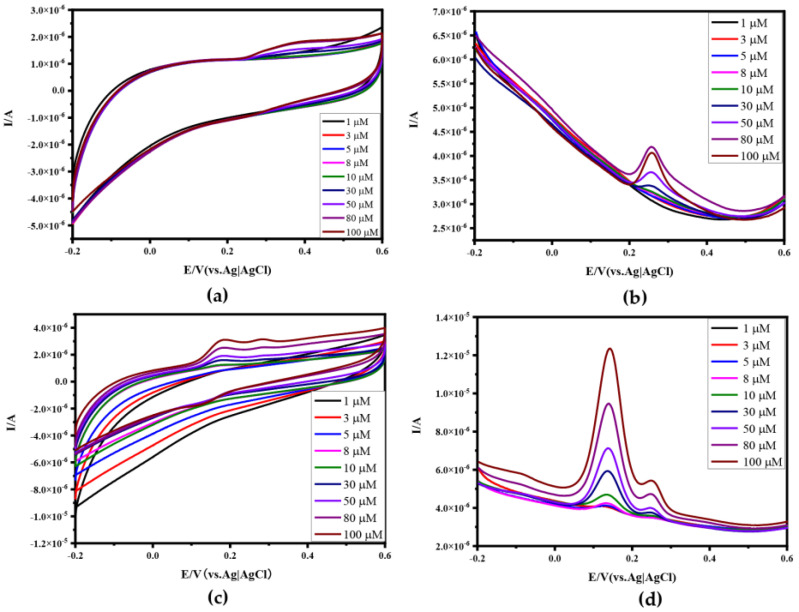
Electrochemical characterization of Apt−Au−N−RGOF in UA solution or mixture solution of UA and DA: (**a**) CV of Apt−Au−N−RGOF in UA solutions with different concentrations (1 μM, 3 μM, 5 μM, 8 μM, 10 μM, 30 μM, 50 μM, 80 μM, 100 μM); (**b**) DPV of Apt−Au−N−RGOF in UA solutions with different concentrations; (**c**) CV of Apt−Au−N−RGOF in mixed solution with different concentrations of DA and UA; (**d**) DPV of Apt−Au−N−RGOF in mixed solution with different concentrations of DA and UA.

In order to further test the stability of Apt-Au-N-RGOF, the stability of Apt-Au-N-RGOF was gradually improved with the increase in the CV times after 100 cycles in the standard blank solution in Figure 6d. From the CV plots of different concentrations of UA from 1 μM to 100 μM, it can be seen that the response to UA is not significant in Figure 7a, and from the corresponding DPV in Figure 5a and Figure 7b, it can be seen that the response of Apt-Au-N-RGOF to detect UA is significantly lower than that of N-RGOF, and there is no oxidation peak when the concentration is lower than 30 μM. However, when the concentration is higher than 30 μM, there is a sharp decline, because at low concentrations, the non-specific recognition molecules cannot be captured by the aptamer, and when the molecules are oxidized, the electron transfer of the non-target molecules is hindered by the aptamer modified on the fiber surface, so the electrons cannot be detected by the microelectrode and cannot produce an oxidation peak. When the concentration of non-target molecules reaches a threshold, the microelectrode detects a low electrical signal. This may be due to the fact that the surface of the microelectrode is not completely covered by the aptamer, and a few molecules directly contact the surface of the microelectrode and transfer electrons to the electrode. The CV and DPV of the mixture of DA and UA are shown in Figure 7c,d. It was found that the aptamer is not affected by the non-target molecules in the mixed solution. The target molecules are detected and the oxidation peak is detected, indicating that the performance of Apt-Au-N-RGOF is better than that of N-RGOF. Apt-Au-N-RGOF further improved sensitivity, selectivity, and anti-interference.

### 3.4. Selectivity, Reproducibility, and Stability of Apt-Au-N-RGOF

**Figure 8:**
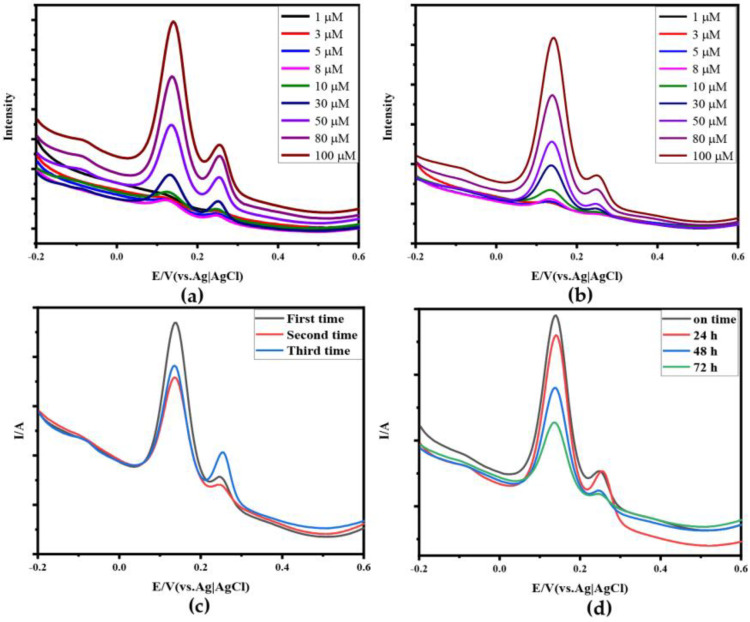
Selectivity, reproducibility, and stability: (**a**) DPV of Au−N−RGOF in a mixture solution of UA and DA with different concentrations (1 μM, 3 μM, 5 μM, 8 μM, 10 μM, 30 μM, 50 μM, 80 μM, 100 μM); (**b**) DPV of Apt−Au−N−RGOF in a mixture solution of UA and DA with different concentrations (1 μM, 3 μM, 5 μM, 8 μM, 10 μM, 30 μM, 50 μM, 80 μM, 100 μM); (**c**) DPV of Apt−Au−N−RGOF in a mixed solution of DA and UA (50 μM) on the first, second, and third time in (**d**) DPV of Apt−Au−N−RGOF in a mixed solution of DA and UA (80 μM) at different times (on time, 24 h, 48 h, 72 h).

From Figure 8a,b, it can be seen that compared to Au-N-RGOF, the Apt-Au-N-RGOF microelectrode can specifically identify and detect DA, avoiding interference from UA and enhancing the selectivity of the microelectrode. In Figure 8c, the reproducibility analysis of the Apt-Au-N-RGOF microelectrode is conducted, showing that after three cycles, it could still specifically identify DA in a mixed solution of DA and UA. In Figure 8d, the stability analysis of the Apt-Au-N-RGOF microelectrode is performed, testing the recognition and detection of a certain electrode on the mixed solution after being placed for different durations.

### 3.5. Electrochemical Response Mechanism of DA

**Figure 9:**
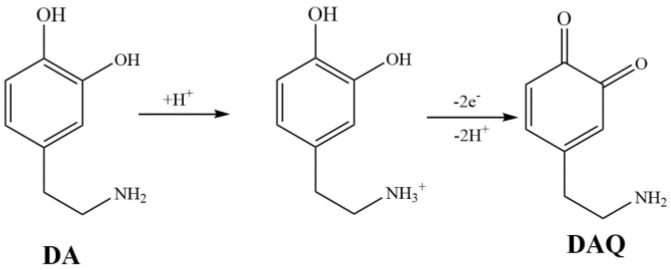
Electrochemical response mechanism of DA.

The oxidation peak potential of DA is fixed at around 0.144 V [6], and the oxidation peak potential of UA is about 0.26 V [7]. In DPV plots, the peak current increases with an increasing concentration. The oxidation mechanism of dopamine involves a two-electron and two-proton transfer process, as shown in Figure 9, where dopamine is oxidized to form dopamine quinone (DAQ). When the electrode accepts electrons from the oxidized dopamine molecules, it generates a Faraday current signal that can be measured, thereby detecting DA [2,7].

## 4. Conclusions

In this thesis, aptamer-modified nitrogen-doped graphene fiber (Apt-Au-N-RGOF) was synthesized and used as a microelectrode to improve the specific adsorption and high-sensitivity detection of target molecules. The electrochemical properties of Apt-Au-N-RGOF- and N-RGOF-modified fibers were tested and compared. We found that Apt-Au-N-RGOF had a higher sensitivity and lower detection limit than N-RGOF. The Apt-Au-N-RGOF microelectrode had a wide linear response to DA in the range of 1 μM~100 μM, with a detection limit of 0.5 μM. From Table 2, based on the comparison with different carbon fibers, Apt-Au-N-RGOF has a higher detection limit and a wider detection range for the mixed solution of DA and UA. The loading of aptamers improves the specific recognition of DA molecules, and the doping of N makes the microelectrode have excellent conductivity. The electrode can be widely applied to the field of electrochemical analysis.

## Figures and Tables

**Table 1:** Element analysis of Au-N-RGOF.

Element	Weight Percentage	Atomic Percentage
C	51.48	80.94
N	3.82	5.16
O	5.53	6.53
Cl	7.83	4.17
Au	30.61	2.93

**Table 2:** Electrochemical detection of DA at different material-modified electrodes.

Materials	Liner Range (μM)	Reference
Apt-carbon fiber microelectrode (CFE)	2–10	[20]
Carbon nanotube/carbon fiber electrode	5–120.6	[23]
AuNPs@NBSAC	1–50	[36]
Apt-Au-N-RGOF	1–100	this work

## Data Availability

Data are contained within the article.
